# Supplementary material for: The Wheat Annexin TaAnn12 Plays Positive Roles in Plant Disease Resistance by Regulating the Accumulation of Reactive Oxygen Species and Callose
Source: Int J Mol Sci. 2023 Nov 16;24(22):16381. doi: 10.3390/ijms242216381 (PMC10671157; doi:10.3390/ijms242216381)
Supplement: Supplementary file 1 [file ijms-24-16381-s001.zip › ijms-2678205-supplementary.pdf]

SUPPLEMENTARY MATERIALS

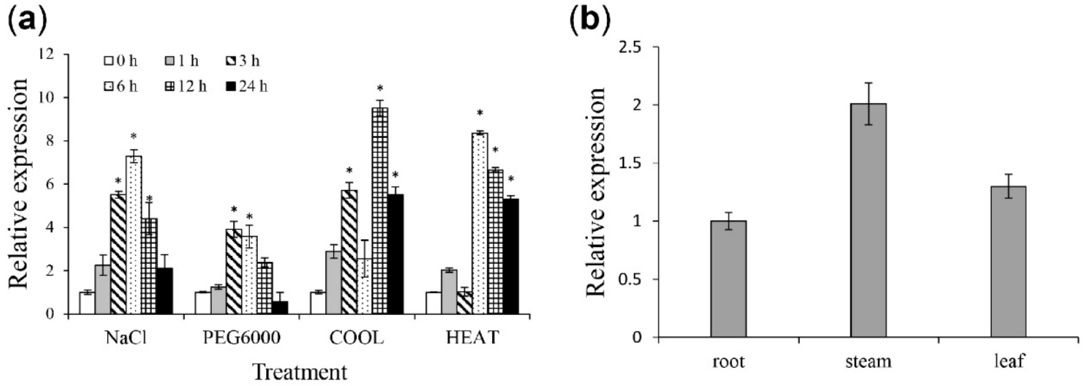

Figure S1: Real-time quantitative PCR (RT-qPCR) analysis of *TaAnn12* expression characteristics. (**a**) The transcription of *TaAnn12* response to abiotic stress. (**b**) Different distribution of *TaAnn12* in pivotal organs. The $2^{-\Delta\Delta t}$ method was used to calculate the relative expression of genes. Error bars represent ± SDs of three biological replications. Asterisks (*) indicate significant differences ($p < 0.05$) from 0 hpi.

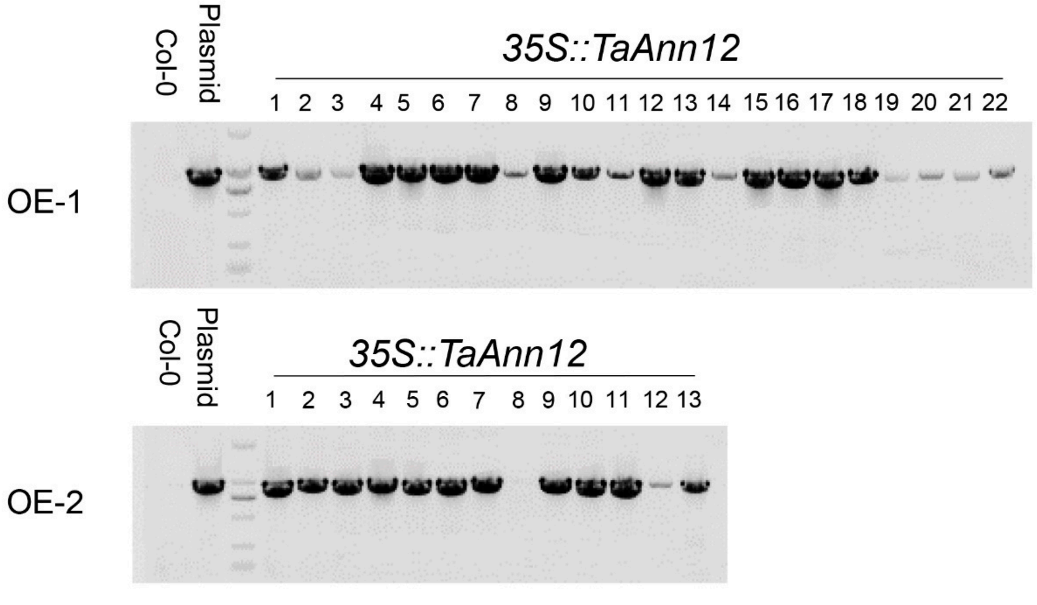

Figure S2: Identification of *TaAnn12* overexpression transgenic plants in T2 generations. Positive *TaAnn12* transgenic plants were identified using PCR.

Table S1. DNA primers used in this study.

Target gene name	Primer name	Sequence (5' to 3')
Gene cloning	TaAnn12-C-F	GGCACTCCATTCGTCCACTC
	TaAnn12-C-R	TCATCGTAAAGCACATCTCT
RT-qPCR analysis	TaAnn12-Q-F	TCTGAAGGCTGACCCCAAG
	TaAnn12-Q-R	GGAGTTCTCATCGGTTCCATT
	TaEF-Q-F	TGCTGTCATCAAGCCTGGTATGGT
	TaEF-Q-R	ACTCATGGTGCATCTCAACGGACT
	TaPR1-Q-F	GAGAATGCAGACGCCCAAGC
	TaPR1-Q-R	CTGGAGCTTGCAGTCGTTGATC
	TaPR2-Q-F	AGGATGTTGCTTCCATGTTTGCCG
	TaPR2-Q-R	AAGTAGATGCGCATGCCGTTGATG
	TaSOD-Q-F	CCGAGGTCTGGAACCATCAC
	TaSOD-Q-R	AGCCGAAATCCTTCTCGATCT
	TaCAT-Q-F	TCGGACACCGAGGACCTATC
	TaCAT-Q-R	CCGTGCATGAACAACACGTT
	TaNPR1-F	CTCAAGGAAGGGCAGACCAAT
	TaNPR1-R	GGCGGCAATGTGAAGAACAGTA
	NQEIF4A-F	CGTAAGCATAGATACCCCTAAGAA
	NQEIF4A-R	TGACCAGAGGCTGAATGAAGT
	AtPR1-F	GTGCCAAAGTGAGGTGTAACAA
	AtPR1-R	CGTGTGTATGCATGATCACATC
	AtPR2-F	CTTAGCCTCACCACCAATGTTG
	AtPR2-R	TCCCGTAGCATACTCCGATTTG
	AtPR5-F	ATGTGAGCCTCGTAGATGGTTAC
	AtPR5-R	GATCCATGACCTTAAGCATGTCG
	AtPDF1.2-F	CTTATCTTCGCTGATCTTGT
	AtPDF1.2-R	CGTAACAGATACACTTGTGTGC
VIGS	TaAnn12-V-F	ccgttgctagctgagcCAGATGGGTCGCAAATGGG
	TaAnn12-V-R	tagctagctgattaatGGAGTTCTCATCGGTTCCATT
Overexpression in Arabidopsis	TaAnn12-O-F	agtccatggtacccgg**ggatcc**ATGGCGACGCTCAAGGTC
	TaAnn12-O-R	cacgggggactctaga**ggatcc**TCACTCCTTCCCCAGGAGG
